# Changes in Quality of Life and Sexual Function After Luteinizing Hormone‐Releasing Hormone (LHRH) Agonists and Orchiectomy in Men With Metastatic Prostate Cancer: Results From a Randomized Trial

**DOI:** 10.7759/cureus.55934

**Published:** 2024-03-11

**Authors:** Niklas Dissing, Mikkel Fode, Peter Østergren, Jens Sønksen

**Affiliations:** 1 Department of Urology, Copenhagen University Hospital, Herlev and Gentofte Hospital, Copenhagen, DNK

**Keywords:** androgen deprivation therapy, urinary dysfunction, erectile dysfunction, quality of life, castration, prostatic neoplasm

## Abstract

Purpose

To examine changes in quality of life (QoL) in men diagnosed with metastatic prostate cancer undergoing androgen deprivation therapy (ADT).

Methods

This was a phase IV trial where patients were randomized to either triptorelin or subcapsular orchiectomy. We report changes in QoL, functional and symptom scales, and sexual function. These were assessed using the validated questionnaires, namely, the European Organisation for Research and Treatment of Cancer (EORTC) Core Quality of Life Questionnaire (EORTC-QLQ-C30), European Organization of Research and Treatment of Cancer Quality of Life Questionnaire Prostate Cancer 25 (EORTC-QLQ-PR25), and Erectile Hardness Scale (EHS) before treatment and at 12, 24, and 48 weeks, respectively. Data were analyzed using linear mixed models for repeated measures.

Results

Fifty-seven men with a median age of 74 years were randomized. The pooled analyses showed that QoL (p=0.003), emotional function (p<0.001), urinary symptoms (p=0.011), and hormonal treatment-related symptoms (p<0.001) changed significantly between visits. Improvement from baseline in QoL (mean change: 6.8 points (95% confidence interval (CI 95% CI): 2.1; 11.5)), emotional function (6.9 points: 3.3, 10.6), and urinary symptoms (-7.7 points (-12.3; -3.0)) was most pronounced at 24 weeks. Hormonal treatment-related symptoms (8.9 points (95% CI: 5.9; 12.0)) worsened. No significant differences between treatment groups were observed. At baseline, 29 men (51%) reported interest in sex, 18 were sexually active, and 12 had erections hard enough for penetration. At 48 weeks seven reported interest in sex, five were sexually active, and one man had a hard enough erection for penetration.

Conclusions

Men with newly diagnosed metastatic prostate cancer experience improved QoL and emotional function after starting ADT. Urinary symptoms improved, while hormonal treatment-related symptoms worsened. Interest in sex and sexual activity was retained in a proportion of men despite ADT.

## Introduction

Prostate cancer is one of the most common cancers among men in the Western world and a leading cause of cancer death. The annual incidence of de novo metastatic prostate cancer is 9.9 per 100,000 men in Denmark [1].

Androgen deprivation therapy (ADT) is the backbone of treatment for metastatic prostate cancer. The aim of ADT is to reduce testosterone levels and thereby control cancer growth. ADT consists of either medical treatment with a gonadotropin-releasing hormone (GnRH) agonist or antagonist or surgical castration. ADT has adverse side effects, which include weight gain, decreased muscle mass, increased insulin resistance, decreased libido, erectile dysfunction, hot flashes, and fatigue [2]. It is known that these side effects often impair the patient's quality of life (QoL) [3].

Treatment options have improved in the last decade for men presenting with de novo metastatic disease. When treated with ADT alone, median overall survival is only around three to five years [4]. Currently, ADT is in most cases combined with other treatments such as radiation of the primary tumor, upfront docetaxel, and/or treatment with a novel androgen receptor-targeted agent [5]. This has led to improved overall survival rates and more men living and coping with the side effects of both ADT and these combination therapies.

We previously conducted a randomized clinical trial (RCT) that compared GnRH agonists with orchiectomy for men presenting with primary metastatic prostate cancer. The primary aim of this trial was to compare metabolic changes. These outcomes have previously been published elsewhere [6,7]. Here, we use data from the same trial to explore changes in specific functional and symptom scales and changes in overall QoL. All outcomes were prespecified before the trial started recruiting.

## Materials and methods

Study design

The data for these analyses were derived from a two-armed RCT among patients with prostate cancer who were starting lifelong ADT. The inclusion period was from September 2013 to March 2015. At this time, ADT monotherapy for de novo metastatic prostate cancer was considered the standard of care. This is the reason why patients did not receive multimodal therapy. The patients were followed over 48 weeks. During treatment, evaluations were carried out after 12, 24, and 48 weeks. 

The patients were randomly allocated to two groups. One group received treatment with the GnRH agonist triptorelin (22.5 mg every 24 weeks), while the other group received a surgical subcapsular orchiectomy. Treatment allocation was determined by a computer-generated randomization sequence, which the investigators were blind to. Following randomization, the investigators were not blinded to treatment allocation. Within 14 days of allocation, both groups began their respective treatments. Patients who were allocated to triptorelin received tablets of bicalutamide (50 mg daily) for the first 30 days to prevent the flare phenomenon.

The study was approved by the Danish Medicines Agency and the Capital Regional Committee on Health Research Ethics in Denmark (H-2-2013-107). The trial was a priori registered at www.clinicaltrialsregister.eu (EudraCT 2013-002553-29). Before inclusion, all patients gave oral and written consent.

Participants

The inclusion criteria were the following: men under 90 years of age, a confirmed prostate adenocarcinoma diagnosis, an Eastern Cooperative Oncology Group performance score ≤2, and indications for lifelong ADT. It was not a requirement that the patients had metastatic prostate cancer on imaging, but this was the primary indication for lifelong ADT. Other indications were patients deemed unfit for curative treatment with high-risk features (i.e., clinically T3/T4 disease and/or PSA > 100 ng/mL and/or regional lymph node metastases (N1)). Patients were staged according to standard practice at our institution with a sodium 18F-fluoride PET/CT and CT of the thorax and abdominal. Few patients had a choline C-11 PET scan or FDG-PET scan. The prostate cancer diagnosis was based on transrectal ultrasound guide biopsis of the prostate. The exclusion criteria were the following: prior pharmacological treatment for diabetes mellitus or osteoporosis, previous androgen therapy, and conditions that would increase the risk of complications to orchiectomy such as hemophilia. Patients who experienced disease progression requiring further treatment and those subsequently diagnosed with diabetes mellitus were excluded from the trial. All patients were enrolled in Herlev and Gentofte Hospital, Denmark.

Outcomes

Changes in QoL, functional and symptom scales, and sexual function were predefined secondary endpoints of the trial and are presented in this article.

The validated European Organization of Research and Treatment of Cancer Quality of Life Questionnaire Core 30 (EORTC-QLQ-C30) and the European Organization of Research and Treatment of Cancer Quality of Life Questionnaire Prostate Cancer 25 (EORTC-QLQ-PR25) were used to evaluate the changes in QoL [8,9]. The Erection Hardness Scale (EHS) was used to evaluate erectile function [10]. The questionnaires were answered on paper at each study visit. Responses to all questions were ensured by the trial personnel before the patients ended the visit. 

Statistical analyses

The statistical analyses were performed using the software R (R Core Team (2016), Vienna, Austria). Sample size determination was not done based on power calculation for changes in QoL, functional and symptoms scales, and sexual function since these were secondary endpoints.

Baseline characteristics and changes in sexual function were analyzed using descriptive statistics. Between-group differences and within-subject changes in QoL, functional, and symptom scales from the EORTC-QLQ-C30 and EORTC-QLQ-PR25 were analyzed with linear regression for repeated measures using generalized least squares. In cases where the patient was excluded from the trial, the value of the last observation was carried forward before the 48 weeks of follow-up. All the tests were two-sided, and the statistical significance level was set to 0.05.

## Results

**Figure 1:**
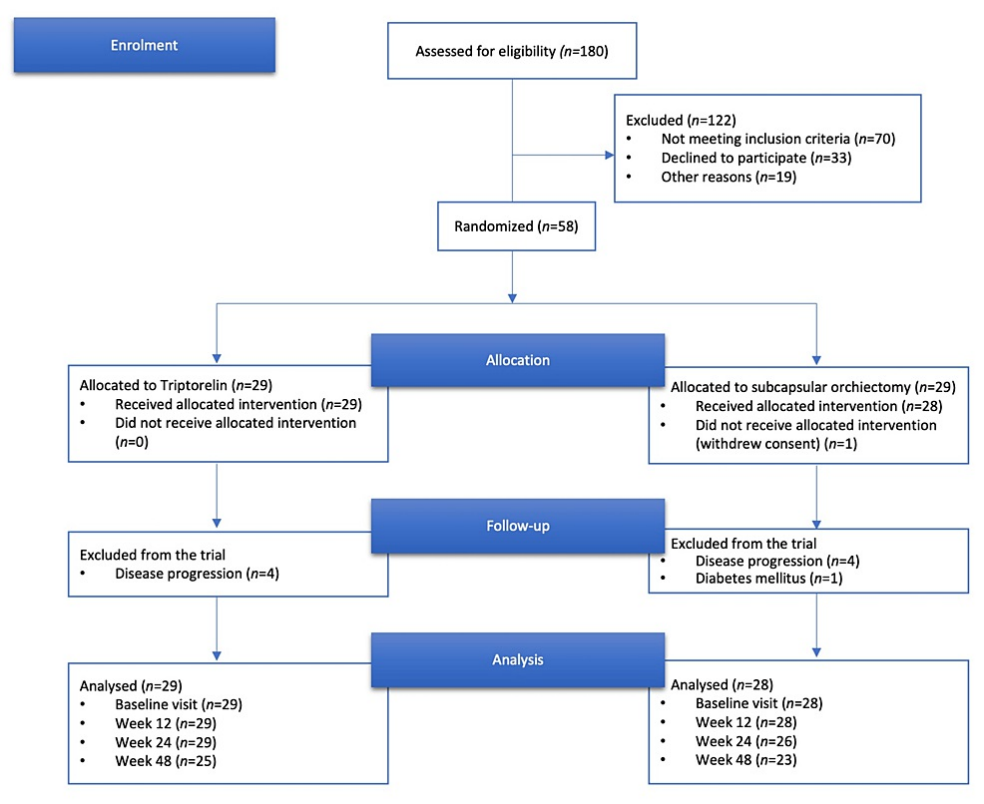
Consolidated Standards of Reporting Trials (CONSORT) study selection flow diagram.

Fifty-seven men with a median age of 74 years (range: 47-86 years) were randomized and received the allocated treatment from September 2013 through March 2015. The majority had metastatic prostate cancer, and none of the patients had prior curative intended treatment (i.e., radical prostatectomy or external radiation beam therapy). The men were randomized to either triptorelin treatment (n=29) or subcapsular orchiectomy (n=28). Baseline characteristics are shown in Table 1. The three follow-up visits were completed by 48 men. Nine men in total were excluded from the trial during follow-up (four men from the triptorelin group and five from the subcapsular orchiectomy group). Eight were excluded due to a progression in their disease, while one was excluded due to a diabetes mellitus diagnosis. The consort flow diagram is shown in Figure 1. Disease progression was patients progressed to having castration-resistant prostate cancer determined at the in-house multidisciplinary conference and based on either new metastatic lesions on imaging or clinical and biochemical progression.

**Table 1:** Baseline characteristics of participants. SD: Standard deviation; PSA: Prostate-specific antigen; IQR: Interquartile range; *mHSPC metastatic hormone-sensitive prostate cancer. Low- and high-volume disease was determined by the CHAARTED criteria (high-volume, four bone metastases with a least one beyond the vertebral bodies and/or pelvis and/or visual metastasis.

	Subcapsular orchiectomy group (n = 28)	Triptorelin group (n = 29)
Age, years mean (SD)	72 (8.8)	75 (5.8)
Body mass index, kg/m^2^ mean (SD)	27.0 (4.8)	27.6 (3.5)
PSA, ug/L median (IQR)	92 (45-352)	61 (37-145)
Clinical T-stage No. (%)		
T ≤ 2	7 (25)	8 (28)
T ≥ 3	20 (71)	20 (69)
Unknown	1 (4)	1 (3)
Regional lymph node metastatic disease, No. (%) (Clinical N stage)		
Present	3 (11)	4 (14)
Not present	25 (89)	25 (86)
Bone and/or visceral metastatic disease, No. (%)	23 (82)	27 (93)
Low volume mHSPC, No. (%)*	11 (39)	15 (52)
High volume mHSPC, No. (%)*	12 (43)	12 (41)
Gleason grading, No. (%)		
< 7	6 (21)	10 (34)
≥ 8	21 (75)	18 (62)
Unknown	1 (4)	1 (3)

The pooled analyses (Table 2), combining data from both groups, showed a statistically significant difference in QoL (p=0.003), emotional function (p<0.001), urinary symptoms (p=0.011), and hormonal treatment-related symptoms (p<0.001) between visits. The difference compared to baseline was most pronounced at 24 weeks with a mean improvement in QoL of 6.8 points (95% CI: 2.1; 11.5). Further, an improvement from baseline in emotional function (6.9 points (95% CI: 3.3; 10.6)) and urinary symptoms (-7.7 points (95% CI: -12.3; -3.0)) was observed at 24 weeks. On the contrary, the hormonal treatment-related symptoms worsened by 8.9 points (95% CI: 5.9; 12.0) at 24 weeks. No statistically significant changes were observed in the other subscales.

**Table 2:** Pooled analyses within-subject changes.

Within-subject changes for pooled analyses (n=57)				
	Mean change from baseline (95% CI)			
	12 weeks	24 weeks	48 weeks	Overall p-value
Quality of life	3.2 (-1.5; 7.9)	6.8 (2.1; 11.5)	3.4 (-1.6; 8.4)	0.003
Physical functioning	-2.6 (-5.7; 0.5)	-3.1 (-6.2; 0.1)	-3.5 (-6.7; -0.3)	0.21
Role functioning	-2.7 (-7.5; 2.1)	-0.6 (-5.9; 4.7)	-0.7 (-6.2; 4.7)	0.67
Emotional functioning	2.3 (-0.9; 5.4)	6.9 (3.3; 10.6)	4.5 (1.1; 7.9)	<0.001
Cognitive functioning	-2.0 (-5.6; 1.6)	-2.4 (-5.8; 1.0)	-2.9 (-6.9; 1.1)	0.54
Social functioning	-2.9 (7.2; 1.4)	-2.2 (-6.0; 1.7)	-3.3 (-7.8; 1.3)	0.51
Fatigue	1.5 (-2.7; 5.3)	2.5 (-1.8; 6.9)	5.56 (0.7; 10.5)	0.10
Nausea and vomiting	0.6 (-2.5; 3.9)	1.0 (-1.8; 3.8)	1.5 (-1.7; 4.7)	0.83
Pain	-4.5 (-9.9; 1.0)	-3.1 (-9.1; 2.9)	-1.0 (-7.5; 5.5)	0.24
Dyspnoea	4.7 (0.2; 9.1)	2.9 (-0.5; 6.3)	1.7 (-0.8; 4.3)	0.06
Insomnia	0.5 (-5.2; 6.2)	1.7 (-5.0; 8.4)	5.3 (-1.9; 12.6)	0.43
Appetite loss	-3.8 (-8.7; 1.0)	-1.4 (-7.6; 4.8)	-0.4 (-5.1; 4.4)	0.40
Constipation	-0.7 (-6.8; 5.3)	-1.2 (-7.6; 5.3)	0.3 (-7.1; 7.7)	0.86
Diarrhoea	-4.1 (-9.0; 0.8)	-5.3 (-9.8; -0.7)	-2.9 (-8.6; 2.8)	0.14
Urinary symptoms	-5.2 (-9.5; -0.9)	-7.7 (-12.4; -3.0)	-6.7 (-11.8; -1.6)	0.010
Bowel symptoms	-1.9 (-3.8; -0.1)	-1.2 (-3.0; 0.5)	-0.6 (-2.5; 1.4)	0.1177
Hormonal treatment-related symptoms	9.3 (6.1; 12.6)	8.9 (5.9; 12.0)	8.6 (6.0; 11.3)	<0.001

No significant differences between treatment groups were observed in any of the scales. The results of between-group analyses are shown in Table 3.

**Table 3:** Between-group differences.

	Subcapsular orchiectomy, mean (SD)	Triptorelin, mean (SD)	Adjusted: between-group difference in ∆ change from baseline, 95% CI (reference=triptorelin)	Overall p-value
Quality of life				
Baseline	71.4 (22.2)	74.7 (19.2)		
12 weeks	73.8 (19.7)	78.7 (18.3)	-3.5 (-11.5; 4.5)	
24 weeks	77.4 (21.4)	82.5 (19.1	-3.4 (-12.0; 5.1)	
48 weeks	72.6 (21.9)	80.2 (20.3)	-5.7 (-15.0; 3.5)	0.66
Physical functioning				
Baseline	88.7 (14.6)	90.8 (17.6)		
12 weeks	85.2 (20.2)	89.0 (15.0)	-2.2 (-8.2; 3.9)	
24 weeks	83.6 (19.9)	89.7 (15.3)	-4.6 (-10.6; 1.4)	
48 weeks	84.8 (18.0)	87.4 (16.7)	-0.95 (-7.2; 5.3)	0.10
Role functioning				
Baseline	83.3 (23.6)	87.4 (27.3)		
12 weeks	79.8 (30.6)	85.6 (25.1)	-2.9 (-12.5; 6.6)	
24 weeks	82.7 (25.0)	86.2 (26.8)	-1.2 (-10.9; 8.6)	
48 weeks	85.7 (26.3)	83.3 (25.2)	4.4 (-5.4; 14.2)	0.37
Emotional functioning				
Baseline	83.0 (19.3)	87.9 (10.8)		
12 weeks	85.1 (16.6)	90.5 (11.3)	-2.6 (-8.1; 2.9)	
24 weeks	91.1 (12.4)	94.3 (10.7)	-1.8 (-7.1; 3.4)	
48 weeks	89.3 (15.4)	91.1 (12.2)	0.3 (-5.4; 6.0)	0.50
Cognitive functioning				
Baseline	89.3 (16.5)	90.8 (10.5)		
12 weeks	86.9 (18.3)	89.1 (11.2)	-1.6 (-8.2; 5.0)	
24 weeks	86.3 (17.6)	89.1 (12.8)	-2.4 (-8.6; 3.9)	
48 weeks	87.5 (19.0)	86.8 (14.4)	1.1 (-6.4; 8.5)	0.38
Social functioning				
Baseline	91.7 (14.7)	96.6 (12.9)		
12 weeks	90.5 (21.0)	92.0 (13.8)	1.3 (-6.8; 9.4)	
24 weeks 48 weeks	89.3 (22.8) 87.5 (24.3)	94.3 (11.2) 94.3 (12.0)	-0.8 (-8.4; 6.8) -3.7 (-12.5; 5.1)	0.59
Fatigue				
Baseline	19.4 (20.4)	15.7 (22.5)		
12 weeks	20.2 (26.0)	17.6 (18.7)	-0.3 (7.9; 7.2)	
24 weeks	21.0 (25.7)	19.2 (23.9)	-1.4 (-10.1; 7.2)	
48 weeks	20.6 (26.1)	24.9 (23.7)	-6.7 (-16.1; 2.8)	0.28
Nausea and vomiting				
Baseline	1.8 (5.25)	2.9 (7.80)		
12 weeks	4.8 (15.6)	1.7 (6.82)	3.0 (-3.0; 9.1)	
24 weeks	5.4 (15.7)	1.7 (6.82)	4.3 (-1.2; 9.7)	
48 weeks	6.6 (19.4)	1.2 (6.19)	6.7 (0.5; 12.9)	0.18
Pain				
Baseline	19.6 (26.9)	8.05 (19.7)		
12 weeks	11.9 (23.1)	6.32 (12.9)	0.1 (-8.3; 8.5)	
24 weeks	12.5 (25.9)	8.62 (14.5)	-1.9(-11.8; 8.0)	
48 weeks	15.5 (26.4)	9.20 (13.1)	2.7 (-7.3; 12.7)	0.69
Dyspnoea				
Baseline	8.3 (17.3)	8.1 (23.0)		
12 weeks	14.3 (23.0)	11.5 (25.6)	2.7 (-6.1; 11.5)	
24 weeks	13.1 (24.6)	9.2 (21.6)	3.8 (-3.0; 10.5)	
48 weeks	10.7 (18.3)	9.2 (23.4)	1.3 (-3.7; 6.4)	0.64
Insomnia				
Baseline	19.0 (29.3)	17.2 (24.6)		
12 weeks	22.6 (31.5)	14.9 (21.1)	6.5 (-4.1; 17.2)	
24 weeks	20.2 (27.7)	20.7 (27.3)	-1.7 (-13.7; 10.3)	
48 weeks	20.2 (30.6)	28.7 (33.0)	-10.9 (-24.6; 2.7)	0.11
Appetite loss				
Baseline	4.7 (14.9)	11.5 (20.5)		
12 weeks	3.6 (13.9)	4.6 (14.7)	1.9 (-5.2; 8.9)	
24 weeks	3.6 (13.9)	10.3 (26.9)	-5.1 (-15.8; 5.5)	
48 weeks	6.0 (15.9)	9.2 (21.6)	1.1 (-7.4; 9.6)	0.33
Constipation				
Baseline	15.5 (23.1)	10.3 (22.0)		
12 weeks	7.1 (16.6)	17.2 (24.6)	-11.0 (-20.3; -1.6)	
24 weeks	13.1 (22.8)	10.3 (18.0)	3.0 (-7.0; 12.9)	
48 weeks	13.1 (24.6)	13.8 (22.7)	-0.4 (-11.8; 11.0)	0.06
Diarrhea				
Baseline	13.1 (18.9)	5.8 (12.8)		
12 weeks	4.7 (14.9)	5.8 (12.8)	-1.8 (-8.7; 5.2)	
24 weeks	4.7 (14.9)	3.5 (10.3)	0.4 (-5.8; 6.6)	
48 weeks	10.7 (25.7)	2.3 (8.60)	7.0 (-2.7; 16.8)	0.39
Urinary symptoms				
Baseline	26.2 (19.6)	26.7 (20.9)		
12 weeks	20.5 (11.4)	21.8 (11.4)	-1.0 (-5.9; 4.0)	
24 weeks	17.5 (12.3)	20.1 (10.4)	-2.6 (-7.9; 2.8)	
48 weeks	20.2 (11.9)	19.4 (10.8)	1.0 (-4.6; 6.7)	0.42
Bowel symptoms				
Baseline	3.6 (6.2)	5.1 (6.8)		
12 weeks	2.1 (4.3)	2.9 (6.0)	-0.3 (-2.8; 2.2)	
24 weeks	2.1 (3.8)	4.3 (6.9)	-1.7 (-4.3; 1.0)	
48 weeks	3.0 (5.7)	4.6 (6.5)	-1.0 (-4.0; 2.1)	0.52
Hormonal treatment-related symptoms				
Baseline	6.9 (8.9)	6.1 (8.6)		
12 weeks	16.3 (12.9)	15.5 (11.0)	0.7 (-5.2; 6.5)	
24 weeks	15.1 (9.5)	15.9 (11.8)	-0.9 (-6.2; 4.5)	
48 weeks	16.1 (9.6)	14.4(9.5)	1.6 (-3.0; 6.1)	0.45

At baseline, 29 men (51%) reported retained interest in sex. Eighteen of the 29 were sexually active. At 12, 24, and 48 weeks, eight, nine, and seven continued to report an interest in sex, respectively, while six, six, and five were sexually active, respectively. At baseline, 12 men had hard enough erections for penetration. This dropped to three men at 12 weeks, while at 24 and 48 weeks, four and one had hard enough erections for penetration, respectively. Only one man used sexual aids, in this case, a PDE-5 inhibitor, to achieve a hard enough erection for penetration.

## Discussion

In this randomized setting, we observed that men diagnosed with primarily metastatic prostate cancer had an improvement in QoL after starting ADT. This finding is contrary to the results of several other studies [3,11-13], which overall show a decrease in QoL after starting ADT. We did not observe a difference in changed QoL, function, or symptom scales between men treated with a GnRH agonist or subcapsular orchiectomy.

The results may be explained by the design of the study and patient group eligible for inclusion and not as a causal effect of ADT. At baseline, the patient group had not received any treatment for their prostate cancer and had received their diagnosis shortly before inclusion. Initiation of known effective treatment is likely to have eased the tension and worry experienced by the patients. Further, at follow-up, the patients are potentially past the crisis following a cancer diagnosis and have come to terms with and gained knowledge about the disease. Both explanations are indirectly supported by the observed improvement in emotional functioning as this is also contrary to findings in other studies. These have shown a worsening in emotional functioning during ADT [14-16], generally explained by the notion that lowering testosterone levels makes patients more emotionally sensitive.

Our results may also be influenced by our patient cohort as all individuals were diagnosed with advanced metastatic disease. When compared to the STAMPEDE trial with upfront abiraterone and upfront docetaxel, it shows that patients in this trial scored numerically lower in baseline QoL [17]. Thus, men with advanced-stage prostate cancer typically experience a higher prevalence of symptoms and, as a result, are likely to report a lower QoL [18,19]. In this context, ADT may help alleviate cancer-related symptoms thereby potentially enhancing patients' QoL [20]. In our patient cohort, the most notable improvements were observed in the management of urinary symptoms. Although these findings did not achieve statistical significance, one could argue that a reduction in pain, less frequent episodes of diarrhea, diminished bowel discomfort, and an increased appetite collectively contributed towards improving the QoL as well.

Concurrently, the lowering of testosterone levels provides an obvious explanation for the worsening of hormonal treatment-related symptoms. The questions used to define the hormonal treatment-related symptoms addressed hot flashes, sore breasts, swelling in legs, and weight loss/gain. These symptoms are known side effects of ADT [14].

Urinary symptoms improved significantly after the patients started ADT. This is consistent with other studies and a known effect of ADT [21]. Urinary symptoms arise when cancer cells grow and proliferate inside the prostate or invade surrounding structures. The cancer cells will then compress the prostatic urethra, urinary bladder, or neurovascular bundles. ADT inhibits the growth and proliferation of cancer cells. Kucway et al. showed a reduction in prostate size by 33% after 3.7 months of ADT [22]. Consequently, a reduction in prostate size will likely lead to fewer urinary symptoms.

At baseline, a proportion of men reported retained interest in sex and were sexually active. For most men, the decrease in testosterone will lead to reduced libido and decreased erectile function [14,23]. However, although the interest in sexual activity, as well as the number of sexually active men, declined after starting ADT, almost a third of the men remained interested in sex. Similarly, Rousseau et al. observed that approximately 20% of the patients were sexually active after receiving ADT for three to six months [24]. In this context, it is important to note that interest in sex and sexual activity is not only hormone-driven. As Fode et al. [25] observed, the main motivation for maintaining interest in sex among men undergoing ADT was the desire for an emotional connection with their partner. The retained interest in sex observed in a proportion of men might be explained by an interplay of psychological and physiological factors beyond having castrate levels of serum testosterone. It is important to be aware and address the patients who retain an interest in sex despite ADT. These patients should be supported and receive relevant advice to ensure they become satisfied with their sex life. Often, this is overlooked during the course of treatment. In our study, the patients, unfortunately, did not receive specific sexual counselling or advice on sexual aids.

Treatment for metastatic prostate cancer has changed since the study was conducted. Nowadays, monotherapy with ADT is rare. ADT is often combined with other treatment modalities, such as treatment of the primary tumor (low-volume metastatic disease), upfront chemotherapy (docetaxel), and/or novel hormone agents (abiraterone, apalutamide, and enzalutamide). The new treatment combinations are observed to improve overall survival and progression-free survival; however, there are more side effects associated with these combinations [26]. Despite more side effects, the combination treatments are not observed to deteriorate QoL. Morgans et al. [27] observed that docetaxel + ADT initially worsened QoL (at three months) compared to patients receiving only ADT. However, the QoL improved after 12 months. The LATITUDE trial reported that abiraterone improved QoL compared to ADT on its own [28]. The same is observed in the ARCHES trial where enzalutamide + ADT is shown to maintain a high-functioning health-related QoL [29].

It should be noted that a potential limitation of the study is the specific criteria used in the selection of patients. At baseline, the patients had just received their cancer diagnosis and had not yet received treatment. Thus, it is likely that the patients were in a crisis and therefore experienced a higher improvement in QoL after starting ADT. Consequently, the results might not be transferable to other stages of the disease. Another potential limitation is the use of the last observation carried forward approach, which positively or negatively affects the results. Only data from 11 time points out of 228 (57 patients with four visits each) were missing, and thus we feel this risk for bias is low. Lastly, QoL was a secondary endpoint to a trial investigating the metabolic impacts of ADT, and the results and conclusions should be seen from this perspective.

## Conclusions

We observed that men with newly diagnosed metastatic prostate cancer starting life-long ADT experienced improved QoL and emotional function after initiation of treatment. This is contrary to the results of several other studies that overall show a decrease in QoL after starting ADT. The improved QoL might be explained by the design of the study as all men were newly diagnosed with primarily metastatic prostate cancer. The patients were potentially past the initial crisis of getting a cancer diagnosis and had come to terms with and gained knowledge about the disease at follow-up. As expected, the men reported improved urinary symptoms, while hormonal treatment-related symptoms worsened after starting ADT. Despite ADT, interest in sex and sexual activity was retained in a proportion of men. Thus, it is important to address this topic in clinical practice and support those patients who retain an interest in sex when undergoing ADT.
